# Correction: Endogenous RNA interference is driven by copy number

**DOI:** 10.7554/eLife.11514

**Published:** 2015-09-25

**Authors:** Cristina Cruz, Jonathan Houseley

Cristina Cruz, Jonathan Houseley. 2014. Endogenous RNA interference is driven by copy number. *eLife* **3**:e01581. doi: 10.7554/eLife.01581

Published 11 February 2014

In the published article, several references were to the wrong articles.

Every time (Akten et al., 2002) was cited in the article, it should have been (Velmurugan et al., 2000).

Every time (Brummelkamp et al., 2002) was cited in the article, it should have been (The ENCODE Project Consortium et al., 2012).

Every time (Caudy et al., 2002) was cited in the article, it should have been (Langmead et al., 2009).

Every time (Han et al., 2002) was cited in the article, it should have been (Derrien et al., 2012).

Every time (Presente et al., 2002) was cited in the article, it should have been (Wong et al., 2002).

Every time (Silva et al., 2002) was cited in the article, it should have been (Hobson et al., 2012).

Every time (Strobel, 2002) was cited in the article, it should have been (Fire et al., 1998).

Every time (Surabhi and Gaynor, 2002) was cited in the article, it should have been (Hannon, 2002).

Every time (Takahashi et al., 2002) was cited in the article, it should have been (Hsieh and Fire, 2000).

Every time (Van Roessel et al., 2002) was cited in the article, it should have been (Gotta et al., 1996).

The article has now been corrected.

